# No Experimental Evidence for Sneaking in a West African Cichlid Fish with Extremely Long Sperm

**DOI:** 10.1155/2013/714304

**Published:** 2013-12-10

**Authors:** Kathrin Langen, Timo Thünken, Theo C. M. Bakker

**Affiliations:** Institute for Evolutionary Biology and Ecology, University of Bonn, An der Immenburg 1, 53121 Bonn, Germany

## Abstract

Alternative reproductive tactics are widespread in fishes, increasing the potential for sperm competition. Sperm competition has enormous impact on both variation in sperm numbers and sperm size. In cichlids, the sperm competition risk is very divergent and longer sperm are usually interpreted as adaptation to sperm competition. Here we examined whether sneaking tactics exist in *Pelvicachromis taeniatus*, a socially monogamous cichlid with biparental brood care from West Africa. The small testis indicates low gonadal investment which is typical for genetically monogamous species. In contrast, sperm length with up to 85 **μ**m is extraordinarily long. We examined the reproductive behaviour of ten groups with a male-biased sex ratio under semi-natural conditions via continuous video recording. We recorded spawning site preferences and correlates of reproductive success and conducted paternity tests using microsatellites. Safe breeding sites that could be successfully defended were preferred. All offspring could be assigned to their parents and no multiple paternities were detected. Body size of spawning pairs predicted their spawning probability and offspring hatching rate suggesting benefits from mating with large individuals. Our study suggests low risk of sperm competition under the given conditions in *P. taeniatus* and thus first evidence for genetic monogamy in a substrate breeding cichlid.

## 1. Introduction

Alternative reproductive tactics (ARTs) are widespread in many animal taxa (e.g., [1, 2]). In particular in externally fertilising fishes there is an enormous potential for ARTs [3–5]. So far, ARTs have been described for more than 170 fish species, 19 of which are cichlids [4]. Due to the diversity of ARTs and fertilisation mechanisms in fishes the potential for sperm competition is high. Sperm competition occurs when sperm of two or more males compete to fertilise a female's eggs [6]. According to sperm competition theory, the strength of sperm competition should influence sperm traits: sperm quantity (e.g., sperm number, usually reflected by testis mass) and quality (e.g., sperm swimming speed [7–11]). The gonadosomatic index (GSI) is generally assumed to be a reliable indicator for sperm competition. It measures gonad mass relative to body mass [12]. Several empirical studies across taxa indeed report that males of polygamous species have a higher relative testis mass (e.g., in birds [13], primates [14], butterflies [15], and fishes [16–18]).

Within species, sperm number is expected to increase in the presence of sneakers to maximise a male's mating success and to outcompete rivals [19, 20]. Three-spined sticklebacks (*Gasterosteus aculeatus*) adjusted their ejaculate size according to sperm competition risk [21, 22]. In the internally fertilising guppy (*Poecilia reticulata*) males with more and faster sperm in the ejaculate reached a greater paternity share [23]. Neff et al. [24] detected a greater ejaculate sperm density in sneaker males than in parental males in the bluegill sunfish (*Lepomis macrochirus*). In the cichlid *Lamprologus callipterus* territorial males ejaculated less sperm than sneakers [25].

Not only sperm number is assumed to be influenced by sperm competition, but also sperm quality like sperm size and motility [11]. Theory predicts a positive relationship between sperm size and strength of sperm competition [7, 26] assuming that longer sperm swim faster and therefore have a higher fertilisation success when competing with sperm of other males [7, 20]. Gomendio and Roldan [27] found evidence for this assumption in mammals in which longer sperm had a higher swimming speed. In the nematode *Caenorhabditis elegans* larger sperm are also faster than shorter sperm [28]. Several studies indeed found a positive relationship between sperm size and sperm competition [29–31]. But in contrast, some studies found a negative relationship (e.g., in old world warblers [32]). Stockley et al. [16] found a negative relationship across fish taxa with polygamous species having shorter sperm than monogamous species.

In cichlids, sneaking is the most common male alternative reproductive tactic in which a male tries to steal fertilisations while a female is spawning with a territorial male (e.g., [3, 33–35]). In some cichlid species, sneaked fertilisations were detected (e.g., [36–38]), while in others no evidence for alternative reproductive tactics was found suggesting genetic monogamy (e.g., [39, 40]). In cichlids, long sperm are usually interpreted as an adaptation to sperm competition and are therefore typical for polygamous species [17]. In East African cichlids, sperm sizes range between 15.5 *μ*m in the monogamous *Asprotilapia leptura *and 33.3 *μ*m in the polygamous *Telmatochromis vittatus* [17]. Fitzpatrick et al. [41] showed that sperm length was positively correlated with sperm swimming speed in Tanganyika cichlids. In cichlids, GSI values are lower than in other fish species [42]. They range from 0.1 to 1.04 in Lake Tanganyika cichlids with polygamous cichlids having a higher GSI [17, 43]. In the polygamous cichlid *Neolamprologus pulcher*, the GSI is around 0.68 [44] and 1.04 in *Telmatochromis temporalis* [43] while in monogamous cichlids the GSI is lower, for example 0.4 in *Tilapia zillii* [45].

A study of Thünken et al. [46] reports an extremely long sperm length of on average 69 *μ*m in the socially monogamous cichlid *Pelvicachromis taeniatus* from West Africa. In comparison with other known sperm lengths of African cichlids, *P. taeniatus* have the longest sperm known in cichlids so far. Opposite to the long sperm length, the GSI in *P. taeniatus* is below 0.2 [47]. A low gonadal investment suggests low sperm competition pointing to genetic monogamy in *P. taeniatus*. In the closely related cichlid *P. pulcher*, three different ARTs occur: monogamous males, harem males, and satellite males [48], with harem males having the highest reproductive success, while dominant satellites are as successful as monogamous males under semi-natural conditions.

The aim of the study was to investigate the reproductive behaviour of *P. taeniatus* under semi-natural conditions. First, we aimed to elucidate whether sneaking tactics occur in *P. taeniatus*. No unambiguous prediction can be made. According to previous studies in cichlids, the low GSI of *P. taeniatus* points to genetic monogamy. On the other hand, the large sperm size points to polygamy and the presence of sneakers. Furthermore, we aimed to examine spawning site preferences and correlates of reproductive success. Here, we predicted greater success of larger males. In fishes, for example, in three-spined sticklebacks (*Gasterosteus aculeatus*), correlations of body size and reproductive success were observed [49]. Laboratory studies in *P. taeniatus* showed that larger males are preferred over smaller males by females as mating partners and larger males are more competitive than smaller ones [50, 51]. Here, we grouped reproductively mature individuals in outdoor enclosures with limited breeding sites increasing the competition between fish. Outdoor enclosures were continuously video recorded and after spawning videos were screened for sneaking events. To detect extrapair paternities, we conducted paternity analyses of clutches using six to ten microsatellites already established for *P. taeniatus* [52].

## 2. Material and Methods

### 2.1. Study Species

*Pelvicachromis taeniatus* is a socially monogamous cave-breeder with biparental brood care that shows size and colour sexual dimorphism [50, 53]. Males defend territories and occupy caves, while females compete with each other for access to males. After spawning, the female cares for the eggs in the cave, while the male defends the territory against intruders [53]. Free swimming fry are guarded by both parents. Pairs stay together for at least one breeding cycle. *P. taeniatus* inhabits small, slow flowing streams within or around woodland. They occupy breeding caves near banks between aquatic plants, branches, roots, and overhanging boundary plants in the shallow water with low flow velocity [54].

### 2.2. Experimental Procedure

In summer 2010, six enclosures (*Ø* 147 cm, 33 cm high, fill level 25 cm, ca. 425 L, INTEX Planschbecken blue, Stans, Switzerland) were positioned outside under a transparent plastic roof at the Institute for Evolutionary Biology and Ecology in Bonn. Preliminary experiments revealed that these enclosures are adequate because at least two occupied territories were established with a territory size similar to those reported for *P. pulcher*, a sister species of *P. taeniatus* showing a similar ecology with territories in nature of about 0.25 m² (see [55]). For thermal insulation, styrofoam plates were positioned under each enclosure and the whole area was enclosed by transparent plastic curtains. Each enclosure was equipped with two internal filters (Dohse Aquaristik, Gelsdorf, Germany), fine sand (ca. 20 L), two heating elements (EHEIM Jäger 250, 400–600 L, Deizisau, Germany), java moss (*Taxiphyllum barbieri*), water milfoil, a mangrove root in the middle of the pool, and two groups each of three flowerpots of different sizes (Ø 6.5 cm, 9 cm, and 11 cm). Differently sized breeding caves were presented in order to investigate whether breeding pairs prefer caves with small entrances which potentially minimise sneaking and egg predation. The water temperature was 24 ± 2°C. Besides the natural daylight, the whole test area was lit for 12 hours by two fluorescent lamps (Lumilux de Luxe daylight, Osram, Munich, Germany, 36 W, from 8 am to 8 pm). Five males and three females of different size and age classes, all reproductively active, were introduced in each enclosure. After spawning, fish and eggs were removed, pools were cleaned, and a new group of fish was introduced.

Before the experiments started, fin clips of 101 individuals of sixteen different F1-families bred from wild-caught *P. taeniatus* from the Moliwe river in Cameroon (West Africa, 04°04′N/09°16′E) were taken and stored in 99.6% ethanol for DNA extraction. Fish were housed in plastic tanks (20 × 30 × 30 cm, 18 L, day length 12 L : 12 D, temperature 25 ± 1°C) equipped with an internal filter, java moss, and fine sand, sorted by family until they were selected and introduced to the pools. For each fish, ten microsatellites were genotyped (see below), and depending on their genotypes, eight fish (five males and three females) of different sizes and ages were selected such that unambiguous assignment of paternities in the group of eight fish per pool was ensured. In both sexes nearly all size classes were present. Females' standard length (SL) ranged from 2.5 to 4.6 cm, total length (TL) from 3.2 to 5.8 cm, and body mass from 0.37 to 2.59 g (mean ± SD: SL = 3.93 ± 0.47 cm, TL = 4.93 ± 0.57 cm, body mass = 1.67 ± 0.55 g). Males' standard length ranged from 3.5 to 8.1 cm, total length from 4.4 to 9.9 cm, and body mass from 0.98 to 11.38 g (SL = 5.71 ± 1.07 cm, TL = 7.18 ± 1.30 cm, body mass = 4.66 ± 2.48). Photos of each individual were taken to be able to distinguish between the eight fish in a pool and to identify breeding pairs according to their dot patterns on caudal and dorsal fins.

Enclosures were digitally video recorded 24 h/d with IP cameras (ALLNET IP Camera, ALL2205 Wireless Indoor, Munich-Germering, Germany), one above each pool. All six cameras were connected to a computer via a switch (ALL8890 8-Port Gigabit Switch ALLNET) and recordings were performed using the IP Surveillance System (ALLNET). Fish were fed *ad libitum* daily with a mixture of defrosted mosquito larvae (*Chironomus*, *Culex *and *Chaoborus*) and *Artemia *in a ratio of 2 : 1 : 0.25 : 1. All caves were daily checked for clutches with an endoscope camera (PX-2235, SOMIKON, Pearl, Buggingen, Germany). After a spawning event had occurred, eggs were removed, counted, and reared in 1 L aerated small plastic tanks (Karlie smart keeper) for five days (pre-tests revealed an adequate DNA concentration in five-day-old larvae) under standardised laboratory conditions (water temperature of 25 ± 1°C; light regime of 12 L : 12 D, Lumilux de Luxe daylight, Osram, Munich, Germany, 36 W). The water was exchanged daily and the hatching rate was determined at the end. For analyses, five-day-old larvae (still having yolk sacs) were transferred in 99.6% ethanol for DNA extraction. Ethanol causes the immediate death of larvae.

After a spawning event, all fish were removed from the enclosure and their body mass and total and standard length were measured. Again, fin clips of all candidate parents were taken and stored in 99.6% ethanol at −20°C. Video recordings were analysed between clutch detection and the cave check the day before (time period on average 26 h 20 min) to look for sneaking attempts during this time period. The spawning pair's frequency of entering and leaving the cave was determined as the number of entering and leaving per hour. Additionally, this frequency was also calculated for the period of spawning (as defined below) and the same period of time before spawning started. The total number of fish that were chased away at the cave was counted before and during the spawning period.

### 2.3. Paternity Assignment

DNA was extracted using the QIAGEN DNeasy Blood and Tissue Kit (QIAGEN). The DNA concentration of each sample was measured using the spectrophotometer NanoDrop 1000 (Thermo SCIENTIFIC) and DNA was adjusted to a uniform concentration of 25 ng/*μ*L with distilled water.

Microsatellites had already been established in *P. taeniatus* through cross-species amplification of microsatellites developed for *Oreochromis niloticus* [56] (see [52]). Additionally, four more loci from Lee et al. [56] were used and one locus from Schliewen et al. [57] (established as described in [52]). Microsatellites were established in a large sample of wild-caught fish (see [52]). Because here we used lab-bred fish out of 16 families, we first tested on polymorphisms and excluded less informative loci. In total ten loci were appropriate for analyses; in most cases six loci were sufficient to determine paternity. If six loci were not sufficient, the four other loci were analysed as well.

The following universal fluorescent dyes and tail primers were used: T7-tail with dye label FAM (5′-[FAM-]TAATACGACTCACTATAG-3′) and Sp6-tail with dye label HEX (5′-[HEX-]GATTTAGGTGACACTAT-3′) according to the tailed primer method by Schuelke [58]. Forward primers were ordered with a tail corresponding to the specific fluorescent labeled primers (T7 tail 5′-TAATACGACTCACTATAG-3′, Sp6 tail 5′- GATTTAGGTGACACTAT-3′). For two loci forward primers were directly labeled: the forward primer of locus GM006 was labeled with FAM and of UNH934 with NED.

PCR reactions were multiplexed with up to three microsatellite loci in one PCR (MIX A: GM006, UNH934, and US758/773; MIX B: GM120, GM658; MIX C: UNH911, UNH855, GM553; MIX D: UNH871; MIX E: UNH971). Amplifications were carried out in a total volume of 10 *μ*L containing 5 *μ*L multiplex mix (Qiagen Multiplex PCR kit, QIAGEN), 1 *μ*L DNA (25 ng/*μ*L), 0.1-0.2 *μ*L forward primer (2.5 pmol/*μ*L), 0.3–0.6 *μ*L reverse primer (5 or 10 pmol/*μ*L), 0.3–0.6 *μ*L labeled primer (5 pmol/*μ*L), and HPLC water. Primer concentrations depended on the strength of locus amplification and dye signal in the multiplexed PCR [59]. PCR amplifications were carried out in a Biometra Tgradient Thermo Cycler (Biometra). The following PCR profile was used: preheating at 94°C for 15 min, 30 cycles of 60 s at 94°C, 45 s at 58°C, 60 s at 72°C, 8 cycles of 60 s at 94°C, 45 s at 53°C, 60 s at 72°C, and a final extension cycle of 30 min at 72°C.

A positive control was run with every PCR batch and a blank sample was included to check for contamination [60]. To calculate the error rate, amplification was repeated with every locus for a subset of 10% of all samples [60, 61] chosen randomly with the RANDBETWEEN function in Microsoft Excel 2007. Afterwards allele sizes were compared and the percentage of mistypes was calculated.

Genotypes were scored on an ABI 3500 (Applied Biosystems). One *μ*L of template was mixed with 0.05 *μ*L of DNA Size Standard 500 LIZ (Applied Biosystems) and 9 *μ*L of HiDi-Formamide (Applied Biosystems).

Alleles were scored with Genemapper version 4.0 (Applied Biosystems). Genotypes of offspring were compared with the genotypes of the potential parents and assigned to the parents to determine paternities and maternities by simple exclusion. Additionally, to double-check, paternity assignment was conducted with Colony version 2.0.1.1 [62] that implements a full-pedigree likelihood method and infers sibship and parentage using the individuals' multilocus genotypes [63]. The mating system was set to female monogamy (no non-territorial female entered the cave) and male polygamy. Inbreeding was inferred because inbreeding occurs in the Moliwe population [52, 53, 64]. The probability that both parents were in the candidate males and females was set to 1. The genotyping error rate was set to the calculated value of the reanalysed samples.

### 2.4. Statistics

All analyses were done with R 2.9.1 [65], given that *P*-values are two-tailed throughout. For one enclosure with larvae the spawning event as well as the breeding cave could not be detected. The spawning pair did not spawn in the flower pots; therefore this clutch was not included in analyses. Body data of males and females, mean total length of spawning pairs [(TL_male_ + TL_female_)/2], clutch size, and hatching rate were correlated using Pearson's tests as data were normally distributed. Paired *t*-tests were conducted to test for differences in the spawning pair's frequency of entering and leaving the cave before and during the spawning event. The chase away frequency was calculated per hour. Generalised linear mixed models (GLMMs) were conducted using the glmm-function in the lme4 package in R. Enclosure was used as random factor in all models. GLMMs were done with a logit link function and a binomial error distribution with spawned/not spawned as dependent variable and total length as fixed factor to test whether there is a relationship between size and spawning. To analyse differences in “approaching” and “chasing away” frequencies between sexes and before and during spawning, GLMMS were done with a log link function with a poisson error distribution using “approach” and “chased away” frequencies as dependent variables, respectively, and sex and before/during spawning as fixed factors. Non-significant factors were removed from analysis and tests of significance were based on likelihood-ratio tests (LRT) following a *χ* ^2^ distribution (see Table 1). All pairs spawned in the smallest flowerpot. We conducted a binomial test to test whether this choice differs from random choice (spawned in smallest cave: yes/no).

## 3. Results

In total, the behaviour of ten groups of fish was recorded. In each pool two breeding caves were occupied each by a mating pair. Nine clutches were found in eight pools, including one pool with two simultaneous clutches of two mating pairs and one pool with larvae. In two pools no spawning occurred. Clutch sizes ranged from 13 to 84 eggs (mean clutch size ± SD = 48 ± 25 eggs). After introduction of fish in the pool it took on average 25  ±  6 days until a pair spawned (*N* = 9). The time of spawning could be narrowed down to a few hours by analysing the video recordings. Usually spawning in *P. taeniatus* consists of a number of sequential spawnings. After the female has glued a few eggs to the ceiling of the cave, the male enters the cave and fertilises the eggs. Then the female starts gluing the next portion of eggs to the ceiling that are then fertilised by the male and so on. The start of the spawning period was determined by the significantly more frequent entering and leaving the cave by both sexes (frequency before the start of spawning: mean = 4.964 ± 4.716, frequency during spawning: 20.383 ± 13.102, paired *t*-test: *t*_7_ = −3.082, *N* = 8, *P* = 0.022). The spawning period lasted on average 3 h 22 min ± 51 min.

**Figure 1:**
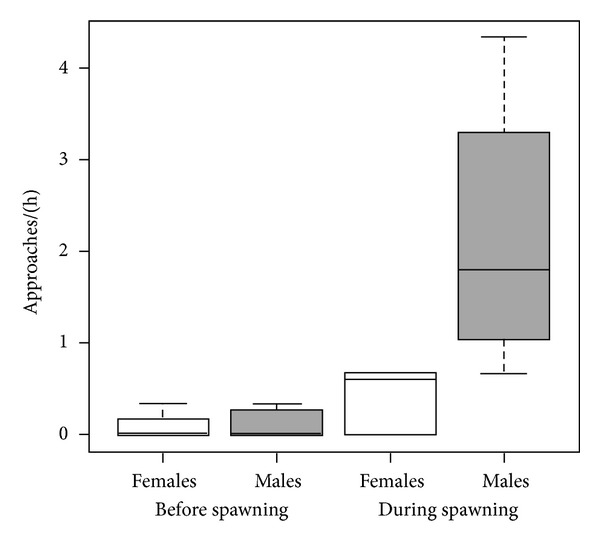
Frequency of males (grey bars) and females (white bars) approaching the cave (median ± quartiles, whiskers) before and during spawning of the territorial spawning pair.

No sneaking events were detected on the video recordings. The cave was intensely guarded by both sexes. Most fish that approached the cave were immediately chased away either by the male or the female, while the other partner stayed inside or outside the cave (mean chase away frequency = 0.925 ± 0.533, *N* = 8). More fish approached the cave during spawning than before (GLMM: *χ*² = 9.021, *N* = 8, *P* = 0.003) with generally more males than females approaching (GLMM: *χ*² = 8.311, *N* = 8, *P* = 0.004) (Table 1, Figure 1). During spawning on average 2.231 ± 1.263 fish were chased away, while before spawning this was on average 0.648 ± 1.484 (GLMM: *χ*² = 6.448, *N* = 8, *P* = 0.011) (Table 1). On average, more males than females were chased away (GLMM: *χ*² = 9.019, *N* = 8, *P* = 0.003) (Table 1). Fish that came close to the cave entrance and tried to enter the cave were immediately chased away (mean chasing away frequency per enclosure = 1.429 ± 0.297; it occurred once before spawning in two enclosures, once after spawning in two other enclosures, and one to two times during spawning in three other enclosures).

**Figure 2:**
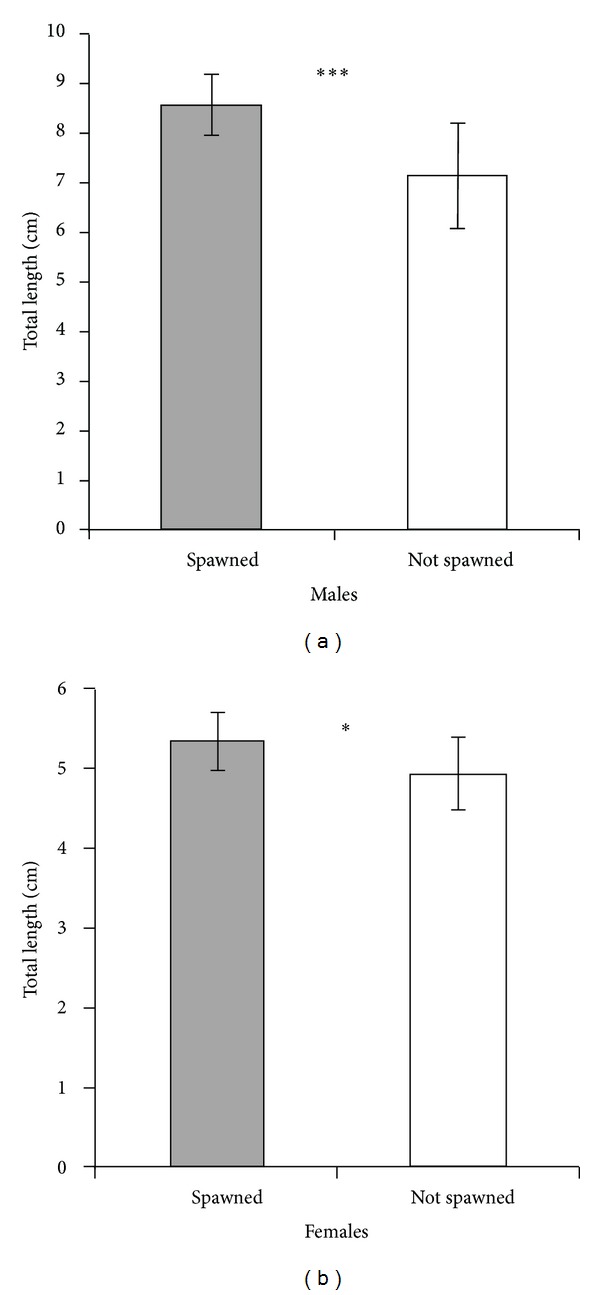
Mean total length (± SD) of spawned and not spawned *P. taeniatus*: (a) males, (b) females. ***P < 0.001, *P < 0.05.

**Figure 3:**
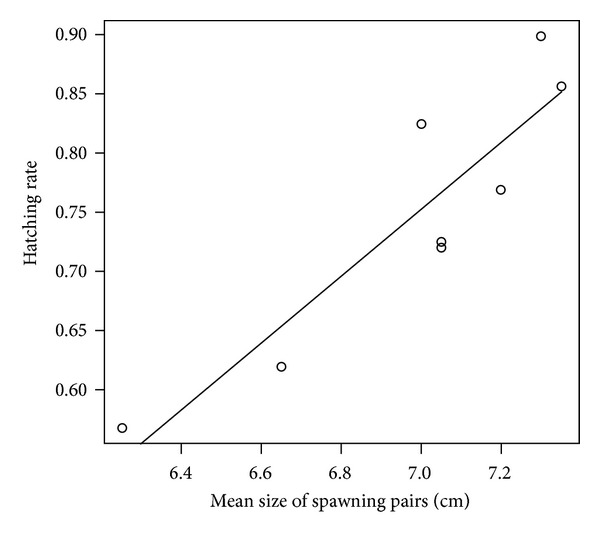
Relationship between mean total length of spawning pairs and hatching rate of the offspring. The line is the least-squares linear regression line.

The video analyses revealed a high activity also during night when fish were often out of their caves and hiding places. However, all spawning events occurred during the day, preferentially during afternoon. Significantly more pairs spawned between 2 pm and 8 pm than between 8 am and 2 pm (*χ* ^2^-test: *χ*_7_ = 4.5, *N* = 8, *P* = 0.034). All 8 pairs spawned in the smallest of the three breeding caves (binomial test: *N* = 8, *P* = 0.008). Successfully mated males were significantly larger than those that failed to spawn (GLMM: *χ*² = 12.554, *N* = 40, *P* < 0.001; Figure 2(a), Table 1); the same was true for females (GLMM: *χ*² = 5.282, *N* = 24, *P* = 0.022; Figure 2(b), Table 1). Mean body size of spawning pairs was significantly positively correlated with fry hatching rate (Pearson correlation: *r*_6_ = 0.807, *N* = 8, *P* = 0.002, Figure 3). There were no significant relationships between body data and clutch size (all *P* > 0.05).

In total, 429 eggs were collected and reared artificially for 5 days of which 327 eggs hatched (hatching rate between 57 to 90%, mean ± SD = 75 ± 11%). Of 327 analysed larvae the total genotyping error rate average was 0.46% (with 2.77% in locus GM006). All offspring could be clearly assigned to their parents using the genotype tables. In all cases, no multiple paternities were detected. Individuals determined as breeding pair with the endoscope camera were also identified as genetic parents. “Colony” revealed the same results with the same individuals as the most likely parents (for all clutches: probability = 1), and all offspring could be clearly assigned. Offspring of one clutch were always full sibs with a probability of 1.

## 4. Discussion

The aim of the study was to investigate whether sneaking tactics exist in the Moliwe population of the West African cichlid *Pelvicachromis taeniatus* using molecular markers for paternity analyses of offspring and continuous video recordings of groups of reproductively active fish in semi-natural enclosures. The results clearly showed a lack of multiple paternities. All produced clutches were sired by the territorial male and female thus indicating genetic monogamy of this population under the conditions tested. This result was expected on the basis of the very low GSI of *P. taeniatus* and suggests that the risk of sperm competition is low in this species. However, on the basis of sperm length, the expectancy is less unequivocal. According to Snook [11] sperm competition influences sperm length in external fertilisers if (a) sperm size affects longevity (positive or negative), if (b) the sperm's competitiveness is determined by swimming speed that is related to sperm length, and if (c) sperm competition intensity is low (i.e., the number of rivals is low). While in Lake Tanganyika cichlids long sperm are typical for polygamous species [17, 41], this does not seem to be the case in the West African riverine cichlid *P. taeniatus*. The co-occurrence of long sperm and monogamy corresponds with the findings of Stockley et al. [16], who showed a negative relationship between sperm competition and sperm length among fishes with polygamous fish species having shorter sperm. So far, sperm swimming speed and sperm longevity are still unknown in *P. taeniatus*. Thus, suggestions about sperm quality cannot be made, but it is possible that *P. taeniatus* produce only few but therefore long sperm of good quality. Further studies in this direction are needed.

Another explanation for the long sperm size in *P. taeniatus* can be that sperm phenotype is haploid controlled [66]. Sperm competition can also occur within sperm of a single male that can lead to the evolution of large sperm in the absence of inter-male sperm competition [66]. Thünken et al. [46] showed a large within-male variation of sperm length in *P. taeniatus* that could point to haploid control of sperm size (see also [67]). Though there is still lacking evidence for haploid selection of sperm and further studies are needed [68]. Large within-male variation may also be indicative of weak selection on sperm length and thus little sperm competition, which further supports the genetic monogamy hypothesis.

Usually monogamous fish species show biparental care of eggs and offspring (e.g., [69–71]) as is the case in *P. taeniatus*. But monogamy and biparental care are rare among fishes [69, 72]. In cichlids, social monogamy was reported in biparental substrate brooders and in some mouthbrooders where males take over larvae from the females' mouth [73, 74]. *P. taeniatus* is a socially monogamous cave-breeder with biparental care as well [64]. There are only few studies on fishes reporting genetic monogamy; examples are the channel catfish *Ictalurus punctatus* [75] that also has very long sperm, seahorses [76], and the largemouth bass *Micropterus salmoides* [70]. In Lake Tanganyika cichlids genetic monogamy was found, for example, in the maternally mouthbrooding cichlid *Tropheus moorii* [40], in the biparentally mouthbrooding cichlid *Eretmodus cyanostictus* [39], and in the mouthbrooding cichlid *Xenotilapia rotundiventralis* [77]. In contrast, a study of Sefc et al. [38] revealed that each of 10 broods is being sired by 2 to more than 10 males in the socially monogamous cichlid *Variabilichromis moorii*. And in the cooperatively breeding cichlid *Neolamprologus pulcher* offspring were sired by at least 2 males in 5 out of 12 groups [34].

Our study was done under conditions simulating those in nature (e.g., concerning territory size [55]) and with a male-biased sex ratio. Under these conditions multiple paternities probably should have been detected if sneaking would be a common tactic in this species. So far, our study reports first evidence for genetic monogamy in a substrate-breeding cichlid but clearly further data from the wild are required to confirm this result.

Spawning occurred during the day but *P. taeniatus* was also very active during night. The high activity during night comes along with studies in new world cichlids that are active at night showing parental care to fan the eggs, attack nest intruders, and care for larvae and fry [78–80]. All breeding pairs chose the smallest caves for spawning. Spawning at daytime and minimising the cave entrance may offer protection against sneakers and thus reduce sperm competition risk but also may protect against egg predation by con- and heterospecifics. Also by mating with a large partner, the competitiveness is increased and territory defence is enhanced. Fish that approached the cave were always chased away and did not get the opportunity to sneak or steal eggs. Although the frequency of approaches was increased during spawning, no males came close to the clutch. The intention of sneaking cannot be ruled out but egg predation is also a possibility. Under laboratory conditions, *P. taeniatus* build sand walls immediately in front of the cave, minimising the size of the entry (KL, personal observation).

Fish that successfully spawned were larger than those that did not. This result underlines previous findings that body size is important in sexual selection and a determinant of reproductive success in fishes (e.g., [81–83]). A study of Baldauf et al. [50] revealed that both sexes of *P. taeniatus* prefer large individuals as mating partners. When given the choice to mate, the probability of mating was higher in pairs with a low size difference leading to size-assortative mating. Furthermore, female standard length was positively related to egg number and significantly to offspring survival. In intrasexual competition larger males seem to have a benefit by outcompeting rival males in contests over breeding caves and therefore may have a higher reproductive success [51]. Our finding of a positive relationship between body size and hatching rate supports these findings.

## 5. Conclusion

In summary, this experimental study provides first evidence for genetic monogamy in a substrate-breeding cichlid fish. The results are in accordance with the low GSI in *P. taeniatus*. By choosing a competitive, large mating partner and a protected breeding site in addition to spawning during daytime breeding pairs probably prevent sneaking.

## Figures and Tables

**Table 1:** Results of the generalised linear mixed models with enclosure as random factor. The dependent variable, explanatory variable, model reduction steps, and test statistics are shown. The effects of female and male body length on spawning are shown. Furthermore, the frequency of approaches of fish to get close to the cave and the frequency of fish that were chased away by the territorial spawning pair depending on period (before/after spawning) and sex were analysed. The sample size (*N*), difference of degrees of freedom (Δdf), and *χ* ^2^- and *P*-values are given.

Dependent variable	Explanatory variable	Δdf	*χ* ^2^	*P*	*N*
Females spawned/not spawned	Total length	1	5.282	0.022	24
Males spawned/not spawned	Total length	1	12.554	<0.001	40

Approaches	Period × sex	1	0.344	0.558	8
	Period	1	9.021	0.003	8
	Sex	1	8.311	0.004	8

Chased away	Period × sex	1	0.275	0.600	8
	Sex	1	9.019	0.003	8
	Period	1	6.448	0.011	8
